# Spinous Process Inclination in Degenerative Lumbar Spinal Stenosis Individuals

**DOI:** 10.1155/2020/8875217

**Published:** 2020-12-15

**Authors:** Janan Abbas, Natan Peled, Israel Hershkovitz, Kamal Hamoud

**Affiliations:** ^1^Department of Anatomy and Anthropology, Sackler Faculty of Medicine, Tel Aviv University, Tel Aviv 6997801, Israel; ^2^Department of Physical Therapy, Zefat Academic College, Zefat 13206, Israel; ^3^Department of Radiology, Carmel Medical Center, Haifa 3436212, Israel; ^4^Azrieli Faculty of Medicine, Bar-Ilan University, Safed 1311502, Israel; ^5^Department of Orthopaedic Surgery, The Baruch Padeh Poriya Medical Center, Tiberias 1520800, Israel

## Abstract

The aim of this study is to determine the sagittal inclination of lumbar spinous processes (SPs) in individuals with degenerative lumbar spinal stenosis (DLSS). It is a retrospective computerized tomography (CT) study including 345 individuals divided into two groups: control (90 males, 90 females) and stenosis (80 males and 85 females. The SP inclination was measured in the midsagittal plane from L1 to L5 levels. Stenosis males (L3-L5) and females (L1, L4) manifested significantly greater SP inclination compared to their counterparts in the control group. Males had significantly horizontal SP orientation compared to females (L1, L2). We also found that SP inclination became steeper as we descend caudally. This study indicates that SP inclinations are significantly associated with DLSS.

## 1. Introduction

Lumbar spinous process (SP) morphometry has become an interesting issue for application in spine surgery. Surgical procedures associated with the SPs are supposed to be easy and quick, with minimal blood loss and soft-tissue disturbance. Previous studies regarding SP morphometry have focused essentially on age-related bony changes [1, 2], gender discrepancy, and the differences between lumbar levels [3–5]. Others have investigated SP biomechanical properties [6, 7] and their morphology regarding the spine devices and surgical techniques [8–10].

Degenerative lumbar spinal stenosis (DLSS) is a common disorder in the elderly population related to degeneration of the spine segment. Additionally, it may narrow the spinal canal and the neural foramen leading to compression of the neural elements [11, 12]. One of the surgical treatments proposed to those with mild clinical presentation of DLSS is the minimally invasive interspinous devices [13, 14]. Although the insertion of these devices depends on the orientation and size of lumbar SPs, few studies have addressed this issue. We also believe that information regarding SP inclination may improve the efficacy of interspinous process devices and shed light on degenerative changes of the posterior elements of the spine.

The aims of this study were (1) to determine the sagittal inclination of lumbar SPs in individuals with DLSS compared to the general population and (2) to establish whether SP inclination is associated with age, gender, and lumbar levels.

## 2. Materials and Methods

### 2.1. Participants

This was a retrospective study including 165 individuals with DLSS-related symptoms who were enrolled between 2008 and 2012. The age range was 40-88 years (sex ratio: 80 M/85 F) with one or more levels with stenosis (cross section area of dural sac < 100 mm^2^) [15]. The diagnostic criteria for DLSS were based on the combination of clinical symptoms and signs together with the computerized tomography (CT) findings [16]. The exclusion criteria were individuals under 40 years of age as well as those with congenital stenosis (anterior posterior diameter of the bony canal < 12 mm) [17], fractures, spondylolysis, tumors, Paget's disease, steroid treatment, severe lumbar scoliosis (>20 degrees), and iatrogenic conditions.

A control group (*n* = 180) without spinal stenosis-related symptoms were referred at the same period to the Department of Radiology, Carmel Medical Center, Haifa, Israel, for abdominal CT scans due to abdominal problems. The age range was 40-99 years (sex ratio: 90 M/90 F).

### 2.2. Computed Tomography (CT) Scans

A high-resolution CT image (Brilliance 64, Philips Medical Systems; voltage: 120 kV; slice thickness: 0.9–3 mm; current: 150–570 mA) was utilized which enabled scan processing in all planes. All the CT images for both groups were taken in supine position with extended knee. This research was approved by the ethical committee of the Carmel Medical Center (0083-07-CMC).

### 2.3. Spinous Process Inclination (SPI)

**Figure 1:**
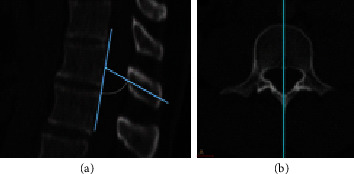
(a) Measurement of spinous process inclination is shown. (b) The location of the midsagittal section of the relevant vertebral body is shown.

Spinous process inclination was measured in the midsagittal plane and defined as the angle between the longitudinal axis of the spinous process and the posterior border of the same vertebral body (Figure 1).

The cross-sectional area of the dural sac [18], lumbar lordosis, and intervertebral disc height [19] were also evaluated in the current study.

### 2.4. Statistical Analysis

The statistical analyses were calculated via SPSS version 20. Independent *t*-test analysis was used for each gender separately to compare between the study groups (stenosis and control) for age, BMI, and SP inclination. One-way ANOVA and Pearson's correlation tests were performed to determine the association between SP inclination and lumbar levels, lumbar lordosis, and disc height, respectively. A logistic regression analysis via the “Forward LR” method (separately for gender) was also used to determine the association between SP inclination and DLSS (dependent variable: DLSS; independent variable: SP inclination, age, and BMI).

The intraclass correlation (ICC) coefficients were calculated to determine the intratester and intertester reliability of the measurements taken (repeated measurements of 20 individuals). Intratester reliability of the measurements was assessed by one of the authors (JA) who took the measurements twice within intervals of 3-5 days. Intertester reliability involved two testers (JA and KH), who took the measurements within an hour of each other. Both testers were blinded to the results of the measurements. Significant difference was set at *P* < 0.05.

## 3. Results

The intratester and intertester reliability results for all the measurements were high: 0.984 to 0.933 and 0.942 to 0.890, respectively.

### 3.1. SP Inclination in the Study Groups (Control vs. Stenosis)

The mean values of age and BMI in the study groups for each gender separately are presented in Table 1.

In all participants, we found that SP inclination angles were significantly greater in the stenosis males (L3-L5) and females (L1-L5) compared to their counterparts in the control group (Table 1). It is noteworthy that this trend was maintained when we did the comparison only for older subjects (>60 year); however, significant differences in females were observed only on the L1 level (Table 1). The outcomes of the logistic regression analysis (adjusted for age and BMI) revealed that more horizontal SP inclinations in males (L3, L5) and females (L1, L4) are significantly associated with DLSS (Table 2*).*

### 3.2. SP Inclination and Age, Gender, Lumbar Levels, Disc Height, and Lumbar Lordosis

Analysis in the control group (*n* = 180) revealed that males had significantly greater SP inclination (more horizontal) than females at the L1 (82.1 ± 5 vs. 79.9 ± 6, *P* = 0.013) and L2 (81.9 ± 6 vs. 80 ± 5, *P* = 0.039) levels.

In general, SP inclinations for males (L1, L2) and females (L3, L5) became more horizontal with increasing age (Table 3). At nearly all lumbar levels, the inclination of SPs was found to be more vertical/steep as we descend caudally; however, the difference achieved significance only for the male group: L2 and L3, L4 and L5 levels (*P* < 0.05). In addition, SP inclination was neither correlated with lumbar lordosis nor with adjacent disc height.

## 4. Discussion

Our results indicate that lumbar SP inclinations (adjusted for BMI and age) for both males (L3, L5) and females (L1, L4) are significantly associated with DLSS. We found that SP inclinations in DLSS individuals were remarkably horizontally oriented relative to the posterior vertebral border compared to the control. Our results also showed that the differences in SP inclination for each gender seem to be age-related variations. For example, the greater differences in SP inclination for males were seen in subjects above 60 years, whereas those for females were supposed to be in subjects between 40 and 60 years old. We believe that this result could support the notion that gender dimorphism affects the prevalence and severity of lumbar spine degeneration [20, 21].

Even though lumbar SP morphology information (e.g., height and width) has been used previously for interspinous process devices, we have found no study in the literature that has addressed the SP inclination in lumbar spinal stenosis individuals. Therefore, others could not confirm our results.

DLSS is correlated with degenerative changes in the three-joint complex and ligamentum flavum thickness [11, 22]. Moreover, the prevalence of this phenomenon increases with age [12, 23]. Intermittent claudication is the main symptom of DLSS that usually worsens in extension and walking and improves in flexion or sitting [24, 25].

The lumbar spinous process is one of the posterior elements of the spine that protects the neural structures in the dural canal and stabilizes the spine unit segment [26]. The interspinous process decompression devices (e.g., XSTOP) provide a minimally invasive technique for DLSS patients with mild symptoms [27]. These devices reduce extension at the symptomatic level(s), intrathecal pressure, and facet load; stretch the ligamentum flavum; and enlarge the neural foramens, thus improving spinal stenosis symptoms [10, 28, 29]. In 2012, Kim et al. have previously reported a strong association between degenerative spondylolisthesis and SP fractures in patients who underwent X-stop devices [9]. The authors believe that the placement of these devices causes destabilization of lumbar segments affected by degenerative spondylolisthesis. One study has recently reported that disc degeneration and fusion intervention correlate with district kinematic alterations of interspinous processes at the involved level [26]. Moreover, spinous process slope has a direct bearing on the efficacy of interspinous process devices to achieve indirect decompression of the canal and neuroforamina [5]. Accordingly, we assume that variations in SP inclination such as horizontal orientation could harm the spine stability due to alterations on the axial loading between the anterior and posterior column.

Our finding in the control group showed that the lumbar SP inclination angle became smaller from L1 to L5. This result is supported by a previous study in which measurements were based on dry skeletal cadavers (2955 human lumbar vertebrae) and found that SP slope became steeper as we descend caudally [5]. We also believe that this result partially supports the notion that the maximal slope of the L5 SP in the general population makes the surgery at the L4 and L5 levels less effective compared to horizontal SPs (L1 through L4) [5].

Similar to the latter study, we also demonstrate significant correlations (on some levels) between SP inclination and age. Although the correlation values are weak (range: 0.221-0.291, *P* < 0.05), it seems that SP inclination became more horizontal in advanced age. We believe that this association could in part be from age-related changes in SP morphology the same as for height and width [1, 2, 30]. With regard to gender, females manifest smaller SP inclination angle at the L1 and L2 levels. Indeed, this trend was noted previously, when Shaw et al. reported that female specimens had generally steeper SP slope than males [5].

It is noteworthy that our SP measurements have some superiority, as the posterior vertebral border (axis reference) is not prone to any age-related changes such as osteoporotic changes of the vertebral body or degenerative changes of the upper end plates that could influence the orientation of the SP, relative to the upper end plate.

Finally, although it is unclear whether the variation of SP inclination in subjects with DLSS is a primary manifestation or secondary process due to aging, we assume that this unique characteristic has a role in the pathogenesis of DLSS. However, the mechanism of SP orientation resulting in DLSS has not been elucidated in detail.

### 4.1. Clinical Implication

As males (L3, L5) and females (L1, L5) with DLSS are candidates for horizontally oriented lumbar SPs, this implies that the efficacy of interspinous devices could be greater at these levels; in addition, spinal/epidural injections could be easier to perform compared with other levels. We also believe that surgeons should take into consideration the fact that SP orientation could be different in subjects with herniated nucleus pulposis, disc disease, lumbar instability, and spinal stenosis when using different interspinous devices [27, 31, 32].

### 4.2. Limitation of the Study

This is a retrospective study, and the outcomes should be supported by well-established prospective studies with large numbers of participants with DLSS. In addition, SP measurements for height, thickness, and width are needed to shed light on the development of DLSS.

## 5. Conclusion

Our results indicate that SP inclinations for both males (L3, L5) and females (L1, L4) are significantly associated with DLSS. Additionally, SP inclinations (on some levels) are dependent for age, gender, and lumbar level.

## Figures and Tables

**Table 1:** Age, BMI, and SP inclination of the study groups (control vs. stenosis) for each gender separately by lumbar level.

Variables	Males		*P* value	Females		*P* value
	Control (mean ± SD)	Stenosis (mean ± SD)		Control (mean ± SD)	Stenosis (mean ± SD)	
All subjects						
Age (years)	62.8 ± 12	66.2 ± 11	0.066	62 ± 12	62 ± 8	0.800
BMI (kg/m^2^)	27.3 ± 4	28.9 ± 4	0.021	27.6 ± 5	31.4 ± 5	<0.001
SP L1 (degree)	82.1 ± 5	81.9 ± 5	0.811	79.9 ± 6	83 ± 4	<0.001
SP L2 (degree)	81.9 ± 6	82.7 ± 5	0.368	80 ± 5	82.4 ± 5	0.006
SP L3 (degree)	78 ± 7	81.6 ± 5	<0.001	77.4 ± 6	80.7 ± 6	0.001
SP L4 (degree)	76.2 ± 7	80.1 ± 6	0.001	76.4 ± 7	80.9 ± 6	<0.001
SP L5 (degree)	72.7 ± 9	76.6 ± 7	0.003	73.3 ± 9	76.5 ± 7	0.017
Subjects > 60 years						
Age (years)	72.9 ± 7	71.6 ± 6	0.284	72.5 ± 8	68.2 ± 6	0.004
BMI (kg/m^2^)	27 ± 4	29.4 ± 5	0.003	26.1 ± 4	31.6 ± 5	<0.001
SP L1 (degree)	82.8 ± 5	82.5 ± 6	0.771	80.3 ± 5	82.7 ± 5	0.034
SP L2 (degree)	82.8 ± 5	83.1 ± 5	0.737	81.1 ± 5	82.1 ± 5	0.429
SP L3 (degree)	79.3 ± 7	82.1 ± 5	0.027	77.9 ± 6	80.5 ± 6	0.052
SP L4 (degree)	76.6 ± 7	81 ± 6	0.003	77.2 ± 7	79.7 ± 6	0.072
SP L5 (degree)	72.18 ± 8	76.5 ± 7	0.009	75.9 ± 8	75.8 ± 8	0.957

SP: spinous process; SD: standard deviation.

**Table 2:** Variables significantly associated with degenerative lumbar spinal stenosis (males and females listed separately).

	OR	CI 95%	*P* value
Males			
BMI	1.097	1.018-1.183	0.015
Spinous process L3	1.087	1.030-1.146	0.002
Spinous process L5	1.061	1.018-1.106	0.005
Females			
BMI	1.159	1.082-1.242	<0.001
Spinous process L1	1.078	1.011-1.150	0.022
Spinous process L4	1.107	1.048-1.168	<0.001

OR: odds ratios; CI: confidence intervals: BMI: body mass index.

**Table 3:** Pearson correlation values between SP inclination and age for each gender separately, by lumbar level.

Variable	Pearson's correlation	*P* value
Males		
Spinous process L1 (degree)	0.211	0.046
Spinous process L2 (degree)	0.187	0.078
Spinous process L3 (degree)	0.213	0.044
Spinous process L4 (degree)	0.123	0.247
Spinous process L5 (degree)	0.015	0.885
Females		
Spinous process L1 (degree)	0.157	0.140
Spinous process L2 (degree)	0.226	0.032
Spinous process L3 (degree)	0.123	0.247
Spinous process L4 (degree)	0.171	0.106
Spinous process L5 (degree)	0.291	0.005

## Data Availability

The data used to support the findings of this study are available from the corresponding author upon request.
